# DNA-PK-Dependent RPA2 Hyperphosphorylation Facilitates DNA Repair and Suppresses Sister Chromatid Exchange

**DOI:** 10.1371/journal.pone.0021424

**Published:** 2011-06-22

**Authors:** Hungjiun Liaw, Deokjae Lee, Kyungjae Myung

**Affiliations:** Genome Instability Section, Genetics and Molecular Biology Branch, National Human Genome Research Institute, National Institutes of Health, Bethesda, Maryland, United States of America; University of Minnesota, United States of America

## Abstract

Hyperphosphorylation of RPA2 at serine 4 and serine 8 (S4, S8) has been used as a marker for activation of the DNA damage response. What types of DNA lesions cause RPA2 hyperphosphorylation, which kinase(s) are responsible for them, and what is the biological outcome of these phosphorylations, however, have not been fully investigated. In this study we demonstrate that RPA2 hyperphosphorylation occurs primarily in response to genotoxic stresses that cause high levels of DNA double-strand breaks (DSBs) and that the DNA-dependent protein kinase complex (DNA-PK) is responsible for the modifications *in vivo*. Alteration of S4, S8 of RPA2 to alanines, which prevent phosphorylations at these sites, caused increased mitotic entry with concomitant increases in RAD51 foci and homologous recombination. Taken together, our results demonstrate that RPA2 hyperphosphorylation by DNA-PK in response to DSBs blocks unscheduled homologous recombination and delays mitotic entry. This pathway thus permits cells to repair DNA damage properly and increase cell viability.

## Introduction

Dividing cells have a higher risk of mutagenesis or death when DNA replication is impeded by exposure to various stresses such as UV or ionizing irradiations and toxic chemicals as well as endogenous cellular byproducts including reactive oxygen species. In particular, unrepaired DNA damage becomes an obstacle for the DNA replication machinery and stalls the progression of DNA replication forks. Stalled DNA replication forks induce checkpoint activation by exposing significant amounts of single-stranded DNA (ssDNA) coated by replication protein A (RPA) due to the uncoupling of the helicase and the DNA polymerases. RPA coated ssDNA recruits checkpoint proteins such as ATR and the 9-1-1 complex (Rad9, Rad1, Hus1) to promote checkpoint activation [1]. One of the most important functions of the replication checkpoint is to stabilize stalled replication forks [2]. Failure of stabilization of stalled replication forks due to the inactivation of ATR or CHK1 leads to forks collapse and exhibits high levels of DSBs [3], [4]. These DSBs could result in hyper-recombination that cause the accumulation of mutations and genomic aberrations [5], [6].

RPA is a heterotrimeric protein complex consisting of three subunits, RPA1 (70 kDa), RPA2 (32 kDa), and RPA3 (14 kDa). RPA2 is differentially phosphorylated during the cell cycle and in response to DNA damage. S23 and S29 of RPA2 are phosphorylated by cyclin dependent kinase (CDK) at the G1- to S-phase transition, and then dephosphorylated at mitosis [7]. In addition, at least 9 RPA2 sites (S4, S8, S11, S12, S13, T21, S23, S29 and S33) are phosphorylated in a complex manner in response to DNA damage and this is usually referred to as “RPA2 hyperphosphorylation” [8], [9]. ATR, ATM, and DNA-PK have all been implicated as the kinase(s) responsible for RPA2 hyperphosphorylation [8], [10], [11], [12], [13], [14], [15], [16], [17]. However, it is still poorly understood whether the hyperphosphorylated RPA2 represents the signal to checkpoint activation or DNA repair and what is the biological function for RPA2 hyperphosphorylation.

Recent studies have demonstrated consequential phenotypes when RPA2 hyperphosphorylation is blocked. Inhibition of RPA2 phosphorylation at T21 and S33 by ATR lead to a defect in delaying DNA synthesis in response to UV damage [10]. Mutations at S23 and S29 in RPA2 caused an abnormal cell cycle distribution both with and without DNA damage [18]. Additionally, these mutations caused persistent staining of γH2AX following DNA damage, suggesting that phosphorylation of RPA2 at S23 and S29 facilitates DNA repair. Interestingly, mutations at S23 and S29 in RPA2 also delayed mitotic exit into G1 phase which was accompanied by a high level of apoptosis in response to bleomycin treatment [18].

Compared to T21, S23, S29, and S33, the residues S4 and S8 of RPA2 have not been studied in as much depth although phosphorylation at these two sites has been used as a marker for the activation of the genotoxic checkpoint due mostly to the logistical availability of an antibody specifically recognizing RPA2 phosphorylated at S4, S8 [19]. Here, we demonstrate that DNA DSBs produced from stalled DNA replication induce S4, S8 phosphorylation in RPA2. Importantly, we also found that DNA-PK, but not ATR or ATM, is the kinase that phosphorylates S4, S8 in RPA2 *in vivo*. RPA2 hyperphosphorylated at S4, S8 delays mitotic entry and appears to prevent unscheduled homologous recombination at collapsed DNA replication forks.

## Results

### RPA2 hyperphosphorylation is induced by DNA damage associated with stalled DNA replication

**Figure 1:**
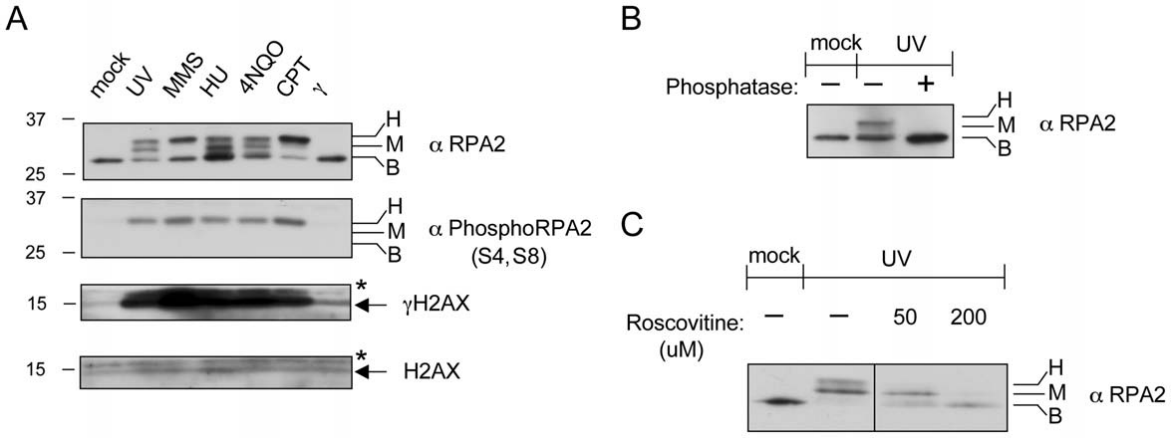
DNA damage that results in DNA replication stalling induces H2AX and RPA2 phosphorylation. (A) Treatment of either 60 J/m^2^ UV irradiation, 0.5 mM hydroxyurea (HU), 2.8 µM camptothecin (CPT), 0.01% methyl methane sulfonate (MMS), or 50 µM 4-nitroquinoline 1-oxide (4NQO) in human HEK293T cells caused H2AX phosphorylation (γH2AX) and RPA2 hyperphosphorylation, whereas 5 Gy of γ-irradiation did not. Cells were treated with the indicated damaging agents for four hours before harvest. (B) Slowly migrating forms of RPA2 are due to phosphorylation. Treatment with λ-phosphatase reduced the slowly migrating forms of RPA2 to the migration position of the unmodified form. (C) Treatment with 50 µM or 200 µM roscovitine for one hour before 60 J/m^2^ UV irradiation in HEK293T cells suppressed RPA2 hyperphosphorylation. Hyperphosphorylation, intermediate phosphorylation, and no phosphorylation of RPA2 are indicated as H, M, and B, respectively.

Previously, we demonstrated that stalled DNA replication induces PCNA ubiquitination [20]. Since RPA accumulates at stalled replication forks [21], we hypothesized that it could be also modified in response to stalled replication. DNA damaging agents, such as hydroxyurea (HU), methyl methane sulfonate (MMS), 4-nitroquinoline 1-oxide (4NQO), camptothecin (CPT), and UV irradiation can cause various DNA damage such as DNA alkylation, DSBs, and DNA crosslinks, and stalled DNA replication forks. HEK293T cells were treated with these DNA damaging agents for 4 hours and the phosphorylation status of RPA2 was subsequently monitored from the chromatin-bound fraction of cell extract. In the UV and γ-irradiation cases, treated cells were allowed to recover for 4 hours and RPA2 phosphorylation was monitored. Similar to PCNA ubiquitination, the phosphorylation of RPA2 was induced in response to HU, MMS, or HU, 4NQO, or CPT treatments, but not to γ-irradiation. Two slower migrating RPA2 bands (marked H for “hyperphosphorylated” and M for “intermediate”) compared to unmodified RPA2 (marked B for “basal”) were detected by Western blotting after these treatments. The slowly migrating forms of RPA2 were due to phosphorylation, because treatment of the extracts with λ-phosphatase before gel electrophoresis eliminated these forms (Fig. 1B). Importantly, RPA2 phosphorylated at S4, S8 was exclusively observed in the H form, but not in the M form (Fig. 1A). Thus, RPA2 hyperphosphorylation can be marked by the specific antibody against S4, S8 phosphorylation of RPA2. We used this specific antibody to detect RPA2 hyperphosphorylation thereafter. Ten Gy of γ-irradiation, a level that is known to cause DSBs without stalling replication did not induce RPA2 phosphorylation when RPA2 phosphorylation was measured 4 hours post-treatment (Fig. 1A). Our results suggest that RPA2 hyperphosphorylation was induced by DNA damage resulting in stalled DNA replication. Interestingly, stalled replication induced by UV, MMS, HU, 4NQO, or CPT causes much stronger intensity of the phosphorylation of H2AX (γH2AX) that marks DNA DSBs than by g-irradiation (Fig. 1A).

### RPA2 hyperphosphorylation depends on CDKs

CDK-dependent phosphorylation of RPA2 at S23, S29 (Figure 1A, M form of RPA2) occurs during DNA replication [19], [22], [23], [24]. To test if RPA2 hyperphosphorylation (Figure 1A, H form) depends on CDKs, HEK293T cells were treated with a CDK inhibitor, roscovitine for one hour to block CDK activities before UV irradiation. Treatment of roscovitine prior to UV irradiation inhibited RPA2 hyperphosphorylation in a dose-dependent manner (Fig. 1C). Therefore, RPA2 hyperphosphorylation in response to UV damage requires the activity of CDKs. Consistently, RPA2 hyperphosphorylation decreases when cells senesce or cells are in a non-dividing status [25].

### DNA-PK hyperphosphorylates RPA2 in vivo in response to UV or 4NQO

Previous studies have implicated various PIKKs responsible for RPA2 hyperphosphorylation, including ATR [10], [11], ATM [15], [16] and DNA-PK [8], [12], [13]. However, the results of these studies were inconclusive in part because various types of DNA damage were investigated, the methods used in these studies to inhibit each PIKK were not very specific, and the phosphorylation sites of different RPA2 molecules could not be monitored specifically. Since the RPA2 hyperphosphorylation was specifically induced by DNA damage stalling DNA replication (Fig. 1A) and an antibody specifically recognizing S4, S8 phosphorylation of RPA2 was available, we investigated which PIKK(s) was responsible for RPA2 hyperphosphorylation.

**Figure 2:**
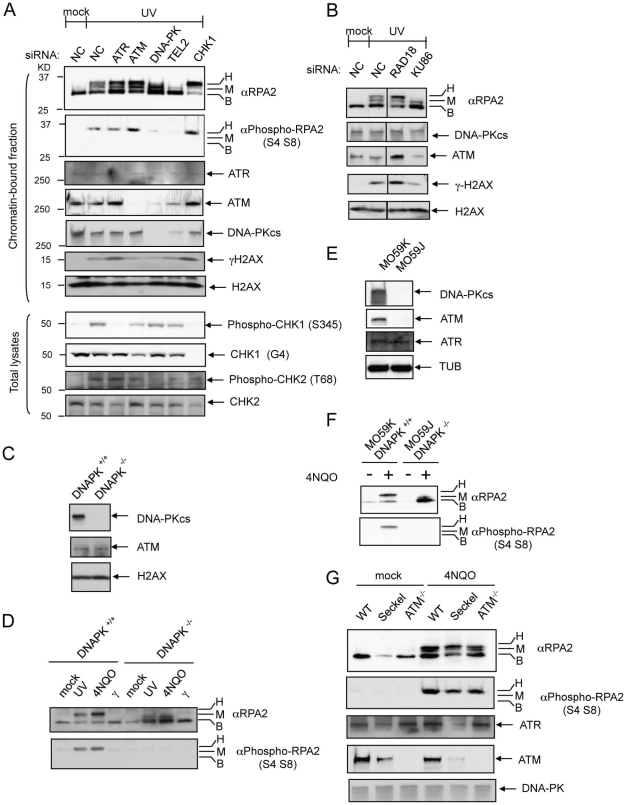
DNA-PK phosphorylates residues S4, S8 in RPA2 in response to DNA damage. (A) RPA2 hyperphosphorylation induced by UV irradiation is dependent on DNA-PK. Expression of ATR, ATM, DNA-PK, TEL2, or CHK1 were silenced by siRNA in HEK293T cells and RPA2 hyperphosphorylation in response to 60 J/m^2^ UV irradiation was monitored. (B) RPA2 hyperphosphorylation is also dependent on the DNA binding subunit of DNA-PK, Ku86. (C) DNA-PK_cs_-null HCT116 cells do not express DNA-PK_cs_. (D) DNA-PK_cs_-null (DNA-PK^−/−^) HCT116 cells do not show RPA2 hyperphosphorylation in response to UV irradiation or 4NQO treatment compared to the parental HCT116 cells (DNA-PK^+/+^). (E) DNA-PK^−/−^ (MO59J) cells do not express ATM and DNA-PK. (F) DNA-PK^−/−^ (MO59J) cells do not show RPA2 hyperphosphorylation in response to 4NQO treatment compared to a matched DNA-PK^+/+^ (MO59K) cell line. (G) Lymphocytes defective in ATR (Seckel) or ATM (ATM^−/−^) as well as wild type lymphocytes produce RPA2 hyperphosphorylation in response to 4NQO treatment. Hyperphosphorylation, intermediate phosphorylation, and no phosphorylation of RPA2 are indicated as H, M, and B, respectively. NC, non-targeting control siRNA.

The expression of ATR, ATM, the DNA-PK catalytic subunit (DNA-PK_cs_), TEL2 or CHK1 was silenced by siRNA (Fig. 2A). Silencing of each gene expression was confirmed by Western blot analysis and qRT-PCR (Fig. 2A and data not shown). Silencing of ATR or CHK1 expression was also verified using a phospho-CHK1 (S345) antibody to confirm the inability of CHK1 to be activated in response to UV (Fig. 2A). As expected, ATM, DNA-PK_cs_, or TEL2 silencing compromised CHK2 activation phospho-CHK2 (T68) in response to UV (Fig. 2A). Importantly, silencing of DNA-PK_cs_ or TEL2 expression almost eliminated the UV-induced phosphorylation at S4, S8 in RPA2 (Fig. 2A, marked H). TEL2 silencing reduced the expression of both ATM and DNA-PK_cs_ (Fig. 2A). Since the depletion of ATM did not cause any reduction in S4, S8 phosphorylation of RPA2 (Fig. 2A, marked H), the reduced S4, S8 phosphorylation of RPA2 by TEL2 silencing appeared to result from the down-regulation of DNA-PK_cs_.

To further investigate the dependency of RPA2 hyperphosphorylation on DNA-PK, RPA2 hyperphosphorylation was examined after silencing the expression of the DNA heterodimeric Ku86∶Ku70 DNA binding subunit of DNA-PK. Similar to DNA-PK_cs_ or TEL2 silencing, Ku86 silencing by siRNA inhibited the hyperphosphorylation of RPA2 in response to UV treatment (Fig. 2B). Consistently, S4, S8 phosphorylation of RPA2 by DNA-PK was observed *in vitro* [19].

DNA-PK dependent phosphorylation at S4, S8 in RPA2 in response to UV treatment was investigated in the DNA-PK_cs_ null HCT116 cell line [26]. In contrast to the normal RPA2 hyperphosphorylation at S4, S8 observed in response to UV or 4NQO treatment in the parental HCT116 cells, RPA2 hyperphosphorylation was completely eliminated in the HCT116 cell line where the DNA-PK_cs_ gene is disrupted by gene targeting (Fig. 2C and D, H form of RPA2). Importantly, primed RPA2 phosphorylated at S23 and S29, which is catalyzed by CDK [19] after UV or 4NQO treatment and required for hyperphosphorylation at the residues of S4 and S8 in RPA2, was detected in DNA-PK_cs_ null HCT116 cells (Fig. 2D, M form of RPA2). Similarly, the M059J cell line that has defects in DNA-PK_cs_ could not induce S4, S8 phosphorylation in response to 4NQO (Fig. 2E and F). In contrast, hypomorphic ATR mutated Seckel cells or ATM null cells (AT) still induced the S4, S8 phosphorylation of RPA2 similar to the wild type in response to 4NQO treatment (Fig. 2G). Taken together, these results strongly suggest that S4, S8 phosphorylation of RPA2 depends on DNA-PK, but not on ATR or ATM.

### The levels of DNA double-strand breaks correspond to hyperphosphorylation of RPA2

**Figure 3:**
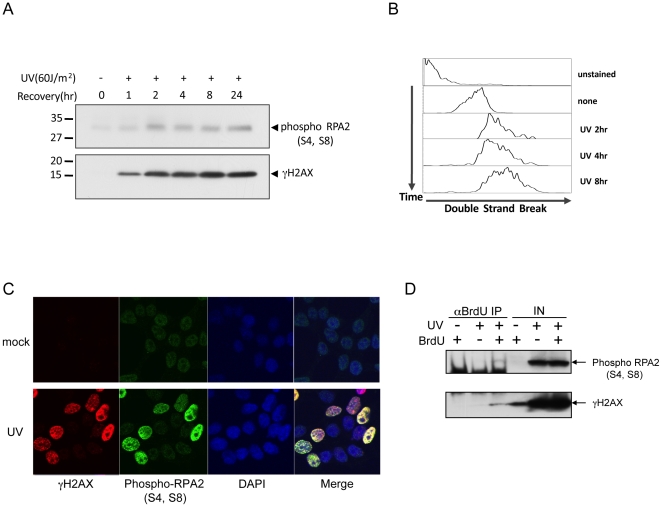
RPA2 hyperphosphorylations correspond to the level of DSBs. (A) Kinetics of RPA2 phsophorylation and γH2AX are similar after UV treatment. HeLa cells were irradiated with 60 J/m^2^ UV and S4, S8 phosphorylation of RPA2 and γH2AX were monitored. (B) DNA DSBs are generated in a similar kinetics with RPA2 phosphorylation after UV treatment. DNA DSBs by TUNEL assay were measured after 60 J/m^2^ UV irradiation using *In situ* Cell Death Detection Kit (Roche). (C) S4, S8 phosphorylated RPA2 foci are co-localized with γH2AX foci in response to UV irradiation. HEK293T cells were stained with specific anti-γH2AX or anti-phospho-RPA2 (S4, S8) (phospho-RPA2 (S4, S8)) antibodies after UV irradiation. (D) S4, S8 phosphorylated RPA2 and γH2AX are enriched at sites of stalled replication. Stalled replication forks that were pulse-labeled with BrdU were then immunoprecipitated with an antibody recognizing BrdU after cross-linking. Proteins in the immunoprecipitate were examined with specific antibodies as indicated.

γH2AX is induced by DNA DSBs [27] and was observed as a consequence of the DNA damage treatments that enhanced RPA2 hyperphosphorylation (Fig. 1A). Therefore, we hypothesized that the DNA damaging agents we had tested were generating DSBs from the collapse of replication forks and such DSBs at forks are substrates for DNA-PK. To explore this possibility, we monitored the kinetics of RPA2 phosphorylation and DSB formation by measuring γH2AX after 60 J/m^2^ UV treatment (Fig. 3A). RPA2 phosphorylation was induced at approximately the same time as when the level of γH2AX was induced. In addition, we measured direct DSBs with a TUNEL assay (Fig. 3B). Similar to the kinetics of γH2AX induction, we could observe DSB formation following UV treatment. Lastly, we analyzed chromosomes from cells treated with UV, HU, 4NQO, or CPT by pulsed field gel electrophoresis. DSBs were markedly visible in cells treated with UV, HU, 4NQO, or CPT with 2.6, 1.7, 9.9, and 3.6 fold more DSBs than the mock treated sample (Fig. S1), which were the same agents that induced RPA2 hyperphosphorylation (Fig. 1A). Interestingly, when cells were treated with high dose of γ-irradiation (e.g. 40 Gy) that generates 2.3 fold more DSBs, we observed the RPA2 hyperphosphorylation at 4 hours post-treatment. This was not present in cells with 5 or 10 Gy of γ-irradiation treatment at 4 hours post-treatment (Fig. 1A and S2). The level of DSB formation by 40 Gy of γ-irradiation was comparable to that of UV, HU, or CPT treatment where RPA2 hyperphosphorylation was observed. In addition, while 5 or 10 Gy of γ-irradiation did not generate enough DSBs to be detected by pulsed field gel electrophoresis and triggered only a mild increase of γH2AX at early time points following 5 or 10 Gy of γ-irradiation, RPA2 hyperphosphorylation as well as a higher level of γH2AX occurred at later times (Fig. S2). Specifically, RPA2 hyperphosphorylation was highly induced at 24 hours post γ-irradiation (10 Gy) exposure. Since 10 Gy of γ-irradiation did not block ongoing DNA replication [20], unrepaired DSBs appear to cause RPA2 hyperphosphorylation when they are processed by resection to produce ssDNA. Therefore, the hyperphosphorylation of RPA2 likely results from high levels of resected DSBs.

To investigate whether the hyperphosphorylated RPA2 localizes to the sites of DSBs, cells were stained with antibodies specifically recognizing phospho-S4, S8 RPA2 and γH2AX before and after exposure to 60 J/m^2^ of UV treatment. Without UV treatment, there was only weak γH2AX and phospho-S4, S8 RPA2 staining (Fig. 3C, upper panel). UV treatment markedly increased the number of cells and the number of foci in the nuclei that positively stained with a γH2AX antibody. Importantly, cells with elevated γH2AX levels were also positively stained with an antibody recognizing phospho-S4, S8 RPA2 (Fig. 3C, bottom panel) and the foci stained by γH2AX co-localized with phospho-S4, S8 RPA2.

Since pulse-labeling cells with BrdU following UV irradiation predominantly labels sites of stalled DNA replication [28], the proteins that can be immunoprecipitated together with BrdU represent proteins that are enriched at stalled DNA replication forks. Since phospho-S4, S8 RPA2 and γH2AX co-immunoprecipitated with a BrdU antibody (Fig. 3D), we inferred that γH2AX and phospho-S4, S8 RPA2 were indeed enriched at the stalled and presumably collapsed replication forks. Taken together, our results suggest that RPA2 hyperphosphorylation corresponds to the level of DSBs generated in cells.

### RPA2 hyperphosphorylation delays mitotic entry

**Figure 4:**
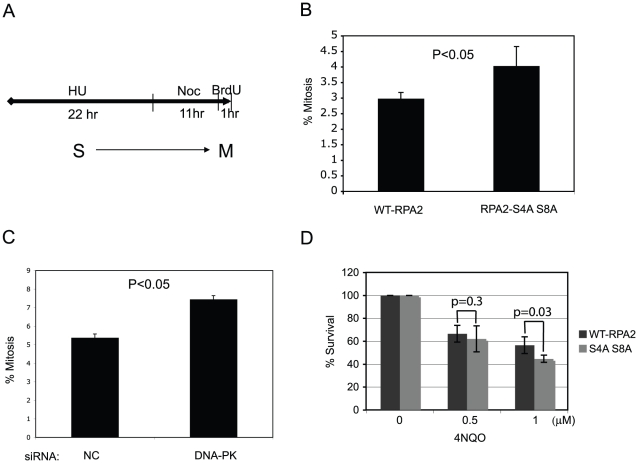
S4, S8 phosphorylation in RPA2 delays mitotic entry and confers resistance to 4NQO. (A) Scheme for HU treatment. (B) RPA2 S4A S8A cells enter mitosis more frequently after release from HU treatment. More than 50,000 cells were counted for each cell type. The average from three independent experiments with standard deviation is presented. (C) Silencing the expression of DNA-PK_cs_ makes cells enter mitosis more frequently after release from HU treatment. More than 50,000 cells were counted for each cell type. The average from three independent experiments with standard deviation is reported. (D) Cells expressing the RPA2 S4A S8A mutant are more sensitive to 4NQO.

Since RPA2 phosphorylation is regulated during the cell cycle and presumably DNA damage should be repaired before entering mitosis, S4, S8 phosphorylation of RPA2 induced by DNA damage could regulate the progression of cell cycle. To examine whether DNA-PK dependent S4, S8 phosphorylation of RPA2 affected the cell cycle, a site-specific RPA2 mutant with S4, S8 changed to alanine (S4A, S8A) was expressed in cells where the endogenous RPA2 had been silenced by siRNA. RPA2 hyperphosphorylation was not detected in the RPA2 S4A, S8A mutant, most notably at S4 and S8 in response to treatment with 2 mM hydroxyurea (HU) for 22 hours (Fig. S3A). In contrast, endogenous RPA2 and wild type transfected RPA2 were hyperphosphorylated by the same stress. Cells expressing exogenous wild type RPA2 (WT-RPA2) or the RPA2 S4A, S8A mutant displayed similar cell cycle profiles in the absence of DNA damaging agents (Fig. S3B). When cells were pre-treated with 2 mM HU for 22 hours to induce the collapse of replication forks (Fig. 4A) and then released into media containing 0.5 µg/ml of nocodazole to inhibit cells from entering another cell cycle, the cells expressing the RPA2 S4A, S8A mutant entered mitosis more frequently than cells expressing the WT RPA2 (Fig. S3C). Independently, we confirmed a higher frequency of cells expressing the RPA2 S4A S8A mutant entering mitosis by counting cells positively stained with phosphohistone H3. There was a significant increase in the population entering mitosis when RPA2 S4A, S8A was expressed after HU treatment; 4% of cells expressing RPA2 S4A, S8A versus 3% of cells expressing WT RPA2 (Fig. 4B and S3D; p<0.05). Similarly, when DNA-PK_cs_ was silenced by siRNA, a significantly higher population entered mitosis (Fig. 4C and S3E; p<0.05). Therefore, DNA-PK-dependent RPA2 phosphorylation at S4, S8 appeared to function in the G2/M checkpoint and caused the delay of cells from entering mitosis with damaged DNA. Consistently, cells expressing the RPA2 S4A, S8A mutant became slightly more sensitive to the DNA damaging agent 4NQO, presumably due to the high level of cell death resulting in cells expressing this mutant protein that subsequently entered mitosis with DNA damage (Fig. 4D). Therefore, at least one of the outcomes of DNA-PK-dependent RPA2 hyperphosphorylation is the delay of mitotic entry, which gives cells more time to repair DNA damage properly.

### RAD51 foci formation and UV-induced sister chromatid exchange rate are increased by expression of the RPA2 S4A, S8A mutant

**Figure 5:**
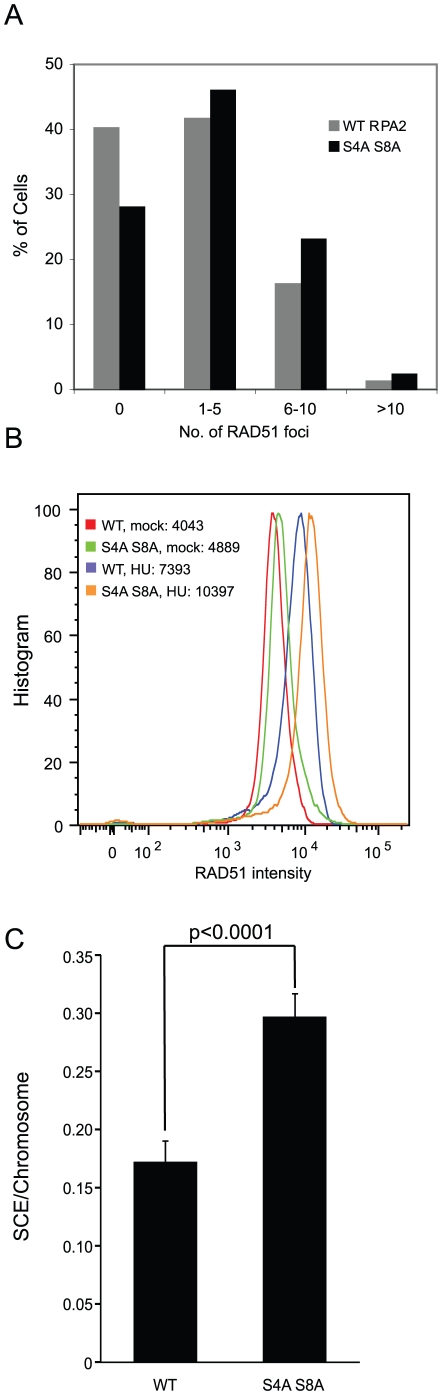
S4, S8 phosphorylation of RPA2 inhibits homologous recombination. (A) Cells expressing the RPA2-S4A S8A mutant protein generate more RAD51 foci than cells expressing wild type (WT)-RPA2. RAD51 foci were counted after cells were treated with 5 Gy of γ-irradiation with 6 hr of recovery. 275 cells of wild type (WT) and 323 cells of mutant (S4A, S8A) RPA2 were analyzed for RAD51 foci formation. (B) Cells expressing the RPA2 S4A, S8A mutant express more RAD51 than WT-RPA2 cells. RAD51 histograms were measured in cells treated with HU for 22 hr or mock treated and allowed to recover for 6 hr. Median numbers are provided. (C) The S4A, S8A mutations cause an increase in the UV-induced SCE rate in HEK293T cells (P<0.0001). Two independent experiments were performed and the average percentage of SCE with standard deviation is reported. A total 66 WT-RPA2 and 89 RPA2 S4A, S8A metaphases cells were counted.

DSBs are often repaired by homologous recombination (HR). HR is initiated by the replacement of RPA with the DNA strand exchange protein RAD51. Given that hyperphosphorylated RPA2 was highly enriched in the chromatin fraction following treatments with DNA damaging agents that caused the collapse of replication forks (Fig. 1), it seemed possible that RPA2 hyperphosphorylation could interfere with HR by affecting RAD51 filament formation. To test this hypothesis, we examined RAD51 foci formation in response to γ-irradiation or HU treatments in cells expressing WT RPA2 or RPA2 S4A, S8A. RAD51 foci formation was induced by γ-irradiation in both WT RPA2 and RPA2 S4A, S8A expressing cells (Fig. 5A and S4). However, when the number of RAD51 foci in individual cells was compared after irradiation, cells expressing RPA2 S4A, S8A had a higher number of RAD51 foci compared to cells expressing WT RPA2 (Fig. 5A). Consistently, the signal intensities of RAD51 were brighter in cells expressing RPA2 S4A, S8A compared to cells expressing WT RPA2 following HU treatment for 24 hours (Fig. 5B). Therefore, blocking S4, S8 phosphorylation of RPA2 enhanced RAD51 foci formation.

The number and intensity of RAD51 foci are closely related with HR frequency [29]. To investigate if the RPA2 S4A, S8A mutation also affects HR frequency, especially in response to DNA damage that results in stalled DNA replication, we measured sister chromatid exchange (SCE) rate in cells treated with 10 J/m^2^ UV irradiation (Fig. 5C). There was a significant increase of SCE rate in cells expressing the RPA2 S4A, S8A mutant protein (p<0.0001). Taken together, the DNA-PK-dependent phosphorylation of RPA2 at S4, S8 appears to block HR and this phosphorylation needs to be removed such that RAD51 foci formation can initiate HR.

## Discussion

In the present study, we demonstrated that RPA2 hyperphosphorylation at S4 and S8 was dependent on DNA-PK. DNA-PK detects DNA DSBs with its DNA end-binding subunit Ku70-Ku80 heterodimer. Similarly, we found that DNA DSBs marked by γH2AX elicited RPA2 hyperphosphorylation by DNA-PK. Higher levels of DSBs generated stronger RPA2 hyperphosphorylation (Fig. 3A). Importantly, DNA-PK-dependent RPA2 hyperphosphorylation requires “primed” RPA2 phosphorylation in other residues of RPA2 that depends on CDK activity. Suppression of CDK activity by roscovitine, eliminates RPA2 hyperphosphorylation (Fig. 1C). Consistently, RPA2 hyperphosphorylation decreases when cells senesce or are in a non-dividing status [25].

Previous studies have shown that ATR, ATM, or DNA-PK can induce RPA2 hyperphosphorylation [10], [15], [16], [30], [31]. However, our results are consistent with several groups that DNA-PK is the major kinase that hyperphosphorylates RPA2 in response to DNA damage [8], [12], [14], [17], [19], [32]. Interestingly, depletion of ATM or ATR did not reduce RPA2 hyperphosphorylation; instead, it enhanced RPA2 hyperphosphorylation. A high level of DSBs generated in ATR- and ATM-defective cells appears to recruit DNA-PK at DSBs to hyperphosphorylate RPA2.

RAD18-dependent post-replication repairs (PRRs) pathways including translesion synthesis and template switching are DNA damage tolerance pathways bypassing DNA damage that results in stalled DNA replications [33]. Although RAD18-dependent PRR does not remove actual DNA damage, it can prevent collapses of stalled forks that can ultimately generate DSBs. Consistently, we observed that RAD18 depletion increased the level of DSBs, as indicated by the increases in both γH2AX and RPA2 hyperphosphorylation (Fig. 2B). Therefore, prolonged stalling of DNA replication due to defects in PRRs appears to result in collapse of DNA replication forks to produce DSBs. Similarly, RPA2 hyperphosphorylation was enhanced in DNA polymerase η-deficient human cells which cannot bypass UV-induced DNA damage [34].

RPA2 hyperphosphorylation depends on DSBs resected to form ssDNAs. In S phase, RPA2 is first primed by CDK-dependent phosphorylation. The primed-phosphorylated RPA is constantly loaded in DNA during DNA replication to cover ssDNA in the lagging strand. Therefore, DSBs generated in S phase already have primed-phosphorylated RPA. In addition, stalled DNA replication forks resulting from DNA damage causes a high level of ssDNA that is rapidly coated with primed-phosphorylated RPA2. Therefore, RPA2 phosphorylation by DNA-PK could be achieved rapidly at stalled replication forks that then collapse into DSBs. In contrast, RPA2 hyperphosphorylation began to appear at 8 hours after exposure to 10 Gy of γ-irradiation (Fig. S2). This delayed RPA2 hyperphosphorylation could be due to the required resection of DSBs to produce ssDNA and loading of RPA with primed-phosphorylated RPA2. Interestingly, an extremely high dose of γ-irradiation (40 Gy) could produce RPA2 hyperphosphorylation in less than 4 hours post-irradiation (Fig. S2). It is puzzling why high dose γ-irradiation can elicit RPA2 hyperphosphorylation in a short time given that primed-phosphorylations in other resides of RPA2 catalyzed by CDK are required for RPA2 hyperphosphorylation. One possibility is that asynchronized populations have enough cells in S phase that have an available supply of primed-phosphorylated RPA2. This supply of primed RPA2 could then be recruited to the numerous 40 Gy-induced resected DSBs and RPA2 hyperphosphorylation could be achieved in a short time. However, the fact that we could not detect any primed-phosphorylated RPA2 in asynchronized cells argues against this possibility. Alternatively, it is possible that RPA2 hyperphosphorylation by γ-irradiation could be different from RPA2 hyperphosphorylation caused by other types of DNA damage.

What is the outcome of RPA2 hyperphosphorylation by DNA-PK? Our results suggest that RPA2 hyperphosphorylation might delay mitotic entry to allow for completion of DNA repair. Therefore, cells expressing the RPA2 S4A, S8A mutant protein that cannot be hyperphosphorylated were sensitive to 4NQO treatment (Fig. 4D). Interestingly, cells expressing the RPA2 S4A, S8A mutant protein enhanced the formation of RAD51 foci, indicating that HR was initiated (Fig. 5A, B). In further support of this, there was a significant increase of SCE in cells expressing RPA2-S4A S8A mutant protein (Fig. 5C). Similarly, HR was suppressed by depletion of protein phosphatase 4, which increases RPA2 hyperphosphorylation or by expression of the RPA2-D4 (S23D, S29D, S33D, S8D) mutant protein, which mimics RPA2 hyperphosphorylation [35]. Therefore, we speculate that DNA-PK dependent RPA2 hyperphosphorylation during DNA replication might inhibit unscheduled HR initiation at collapsed forks.

We propose that DNA-PK is recruited to DSBs during DNA replication and hyperphosphorylates RPA2 to give cells chance to complete DNA repair before mitotic entry [9], [10]. At the molecular level, DNA-PK dependent RPA2 hyperphosphorylation appears to suppress unscheduled HR that might lead to genomic instabilities including translocations and genomic rearrangements.

## Materials and Methods

### Cell Culture, Reagents, and Antibodies

HEK293T cells were cultured in DMEM supplemented with 10% fetal bovine serum (FBS), and 2% glutamine. U2OS/DR-GFP cells were grown in McCoy's media supplemented with 10% FBS. The antibodies used were anti-RPA2 (Calbiochem), anti-phospho-RPA2 (S4, S8) (Bethyl Laboratories), anti-phospho-RPA2 (S33) (Bethyl Laboratories), anti-ATR (Santa Cruz), anti-ATM (Santa Cruz), anti-DNA-PK_cs_ (Thermo Scientific), anti-CHK1 (Santa Cruz), anti-CHK2 (Santa Cruz), anti-phospho-CHK1 (Ser 345) (Cell signaling), anti-phospho-CHK2 (Thr 68) (Cell signaling), anti-γH2AX (Upstate), anti-H2AX (Abcam), anti-HA (HA-7, Sigma). The SMART pools of all gene-specific siRNAs were purchased from Dharmacon. MMS, HU, 4NQO, camptothecin were purchased from Sigma.

### Construction of Various Expression Plasmids

Both wild type RPA2 and the S4A S8A mutant RPA2 used in this study contain an HA epitope tag. HA-RPA2 S4A, S8A was generated by site-directed mutagenesis (Stratagene) with two primers GAC CAA GAT GTG GAA CGC TGG ATT CGA AGC CTA TGG CAG CTC CTC and GAG GAG CTG CCA TAG GCT TCG CCA GCG TTC CAC ATC TTG GTC.

### Detection of Chromatin-bound RPA2

HEK293T, MO59K, MO59J, HCT116 (a gift from Dr. Eric Hendrickson's laboratory, University of Minnesota), Seckel, or AT cells (purchased from Coriell Institute) (as shown in Figure 1 and 3 respectively) were treated with DNA damaging agents for four hours before the cells were harvested as indicated in figures. When cells were treated with either IR or UV, cells recovered for four hours after irradiation and were then harvested. To make the chromatin-bound fraction, approximately 10^7^ cultured cells were resuspended in buffer A (10 mM HEPES (pH 7.9), 10 mM KCl, 1.5 mM MgCl_2_, 0.34 M Sucrose, 10% Glycerol, 1 mM PMSF, 5 µg/mL aprotinin, 20 µg/mL leupeptin) and Triton X-100 was added (to a final concentration of 0.1%). Precipitated chromatin-bound fractions obtained by centrifugation at 4°C at 1,300×g for 5 min were then resuspended in TSE500 buffer [20 mM Tris (pH 8.1), 2 mM EDTA, 500 mM NaCl, 0.1% SDS, 1% Triton X-100, protease inhibitor cocktail (Roche)] and sonicated. Chromatin-bound proteins were collected from the supernatant after centrifugation at 17,000×g for 5 min.

### RAD51 Immunofluorescence Microscopy

10^5^ HEK293T cells plated on two-well chamber slides were transfected with siRNA specifically targeting the 3′-UTR of RPA2 (Dharmacon, SMART pool, #9, #11, #12), together with a plasmid expressing either siRNA-resistant HA-tagged RPA2 or RPA2 S4A, S8A and cultured for three days. The cells were then irradiated with 5 Gy of γ-irradiation followed by a 6 hr recovery period and then fixed with 3.5% paraformaldehyde for 15 min and permeabilized with 1% Triton X-100 for 10 min. Fixed cells were blocked with 5% FBS and stained with anti-RAD51 and rhodamine-conjugated anti-mouse secondary antibodies.

### Flow Cytometry Analyses

Two days post transfection (to allow cells to replace the endogenous RPA2 in HEK 293T cells as described above), cells were incubated with 2 mM of HU for 22 hr. Cells were then cultured in fresh medium without HU but with 0.5 µg/ml of nocodazole for 12 hr. BrdU was added at 1 hr before harvest. Cells were then fixed and stained with both APC-conjugated anti-BrdU and FITC-conjugated anti-phospho-H3 antibodies. Cells were sorted by Becton Dickinson FACSCaliburs as described [20].

### siRNA and Recombination Reporter Assay

U2OS human osteosarcoma cell lines stably transfected with a single copy of an intact DR-GFP reporter gene were used to measure the HR frequency [36]. The endogenous RPA2 was replaced with HA-tagged RPA2 or RPA2 S4A, S8A as described above. Two days later, a plasmid pCAG-I-SceI (which expresses I-SceI enzyme), or an empty vector, pCAG, together with a pDsRed monomer (as a transfection efficiency control) were transfected. HR frequency was determined by the number of cells expressing GFP divided by the number of cells expressing DsRed monomer. Experiments were repeated at least three times and the average values are reported.

### Pulsed field Gel Electrophoresis

∼2.5×10^6^ of HEK293T cells were treated with DNA damaging agents for 4 hr. Cells were then trypsinized and melted into SeaPlaque GTG agarose (Cambrex Bio Science Rockland, Inc) with 0.75% agarose final concentration. Agarose plugs were then digested in proteinase K reaction buffer (100 mM EDTA, pH 8.0, 0.2% sodium deoxycholate, 1% sodium lauryl sarcosine, and 1 mg/ml Proteinase K) at 50°C for 2 days and washed 4 times in wash buffer (20 mM Tris, pH 8.0, 50 mM EDTA). The plugs were loaded onto a 1% pulsed field certified agarose (Biorad). Separation was performed on a CHEF-DR III pulsed field electrophoresis systems (Biorad; 120 field angle, 240 s switch time, 4 V/cm, 14°C) for 24 hr. Gels were stained with ethidium bromide and DSBs were quantified and analyzed using fluorescence image analyzing system FLA 5100 (FujiFilm).

### Cell Survival Assay

Cell viability was determined in HEK293T cells where endogenous RPA2 was replaced by WT-RPA2 or RPA2 S4A, S8A. Approximately 6000 cells were plated in triplicate on 96-well plates. 4NQO was added into the medium16 hr after plating. Cell viability was determined three days later by the CyQUANT Cell proliferation assay kit (Invitrogen).

## Supporting Information

Figure S1 **DNA damaging agents that stall DNA replication cause high level of DSBs.** Chromosomal DNAs isolated from HEK293T cells treated with various DNA damaging agents were separated by pulse-field gel electrophoresis. The levels of DSBs were normalized to that in the no treatment lane (mock).
(TIF)

Figure S2 **DSBs as well as RPA2 hyperphosphorylation were increased when cells were incubated after treatment of γ-irradiation.** Chromatin-bound fractions from HEK293T cells irradiated with the indicated doses of γ-irradiation were prepared at different time points (10 Gy) or four hours after irraditation (40 Gy) and phosphorylation of H2AX (γH2AX) and RPA2 hyperphosphorylation were examined. Hyperphosphorylation, intermediate phosphorylation, and no phosphorylation of RPA2 are indicated as H, M, and B, respectively.
(TIF)

Figure S3 **S4A S8A mutations in RPA2 cause higher frequency of mitotic entry.** (A) RPA2 S4A, S8A mutant is not hyperphosphorylated in response to DNA damage. Cells were treated with HU in condition described in “*C*”. B1 and B2 are unmodified endogenous and transfected HA-tagged RPA2, respectively. H1 and H2 are hyperphosphorylated endogenous and transfected HA-tagged RPA2, respectively. (B) Cells expressing either wild type RPA2 or S4A S8A mutant RPA2 (S4A S8A) did not show any distinct differences in cell cycle profiles. (C) Cells expressing S4A S8A mutatant RPA2 entered mitosis more frequently after release from DNA replication stress by HU. Cells were treated with HU for 22 hours and washed to release into media having Nocodazol without HU to inhibit another round of cell cycle. After 11 hours incubation, cells were pulse-labeled with BrdU for one hour before FACS analysis. (D) Mitotic cells positive in both BrdU and Phospho H3 were increased in cells expressing S4A S8A RPA2 mutant. (E) Silencing the expression of DNA-PKcs increased mitotic cells that were positive in both BrdU and Phospho H3.
(TIF)

Figure S4 **S4A S8A mutations in RPA2 increased the number of RAD51 foci in response to 10 Gy γ-irradiation.**
(TIF)
