# Supplementary figures and images for: DNA-PK-Dependent RPA2 Hyperphosphorylation Facilitates DNA Repair and Suppresses Sister Chromatid Exchange
Source: PLoS One. 2011 Jun 22;6(6):e21424. doi: 10.1371/journal.pone.0021424 (PMC3120867; doi:10.1371/journal.pone.0021424)

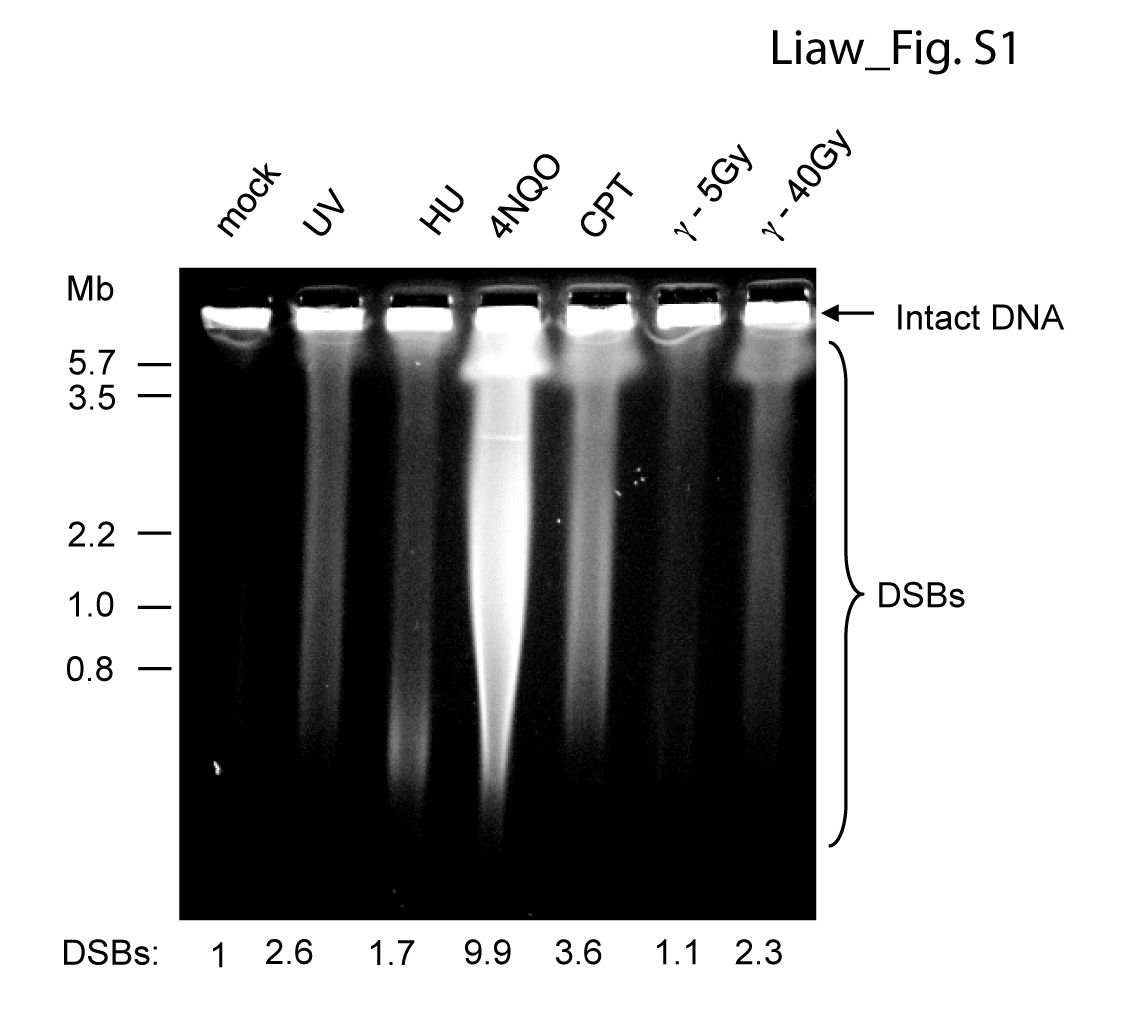

Supplement: Figure S1 — DNA damaging agents that stall DNA replication cause high level of DSBs. Chromosomal DNAs isolated from HEK293T cells treated with various DNA damaging agents were separated by pulse-field gel electrophoresis. The levels of DSBs were normalized to that in the no treatment lane (mock). (TIF) [file pone.0021424.s001.tif]

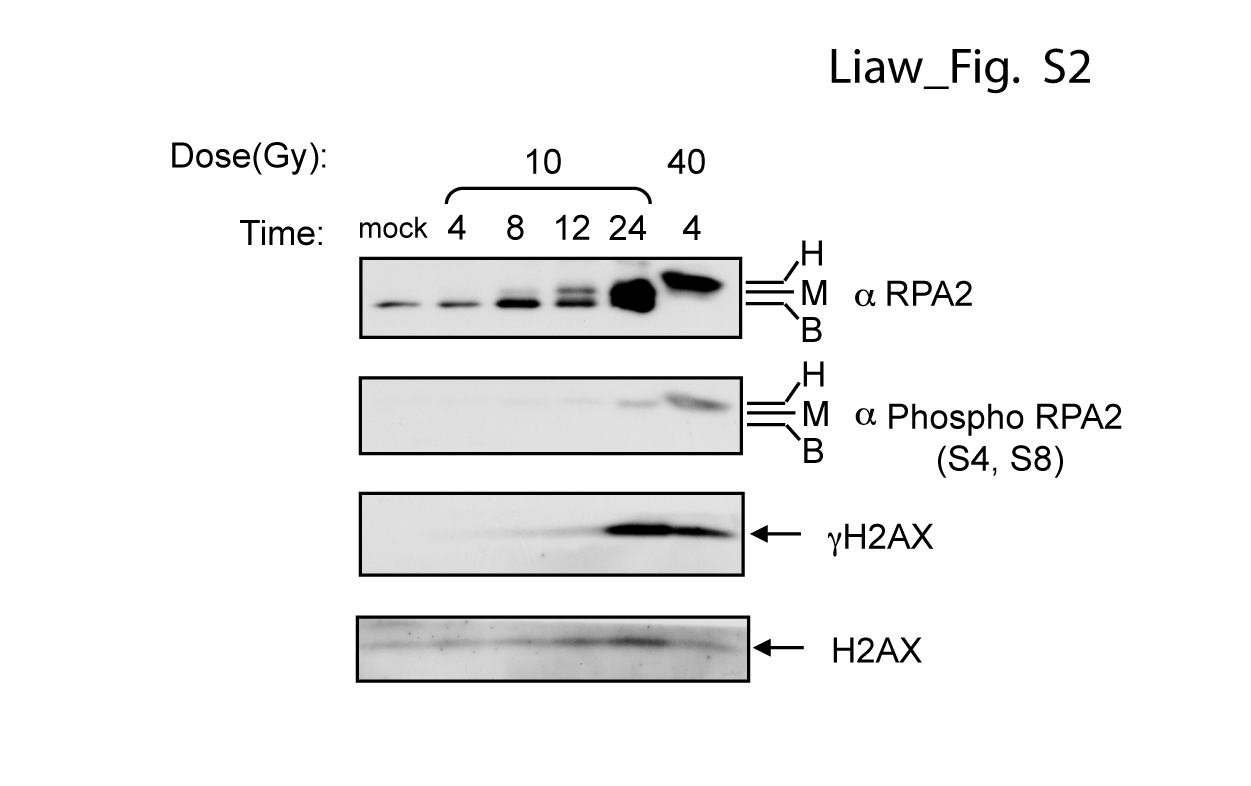

Supplement: Figure S2 — DSBs as well as RPA2 hyperphosphorylation were increased when cells were incubated after treatment of γ-irradiation. Chromatin-bound fractions from HEK293T cells irradiated with the indicated doses of γ-irradiation were prepared at different time points (10 Gy) or four hours after irraditation (40 Gy) and phosphorylation of H2AX (γH2AX) and RPA2 hyperphosphorylation were examined. Hyperphosphorylation, intermediate phosphorylation, and no phosphorylation of RPA2 are indicated as H, M, and B, respectively. (TIF) [file pone.0021424.s002.tif]

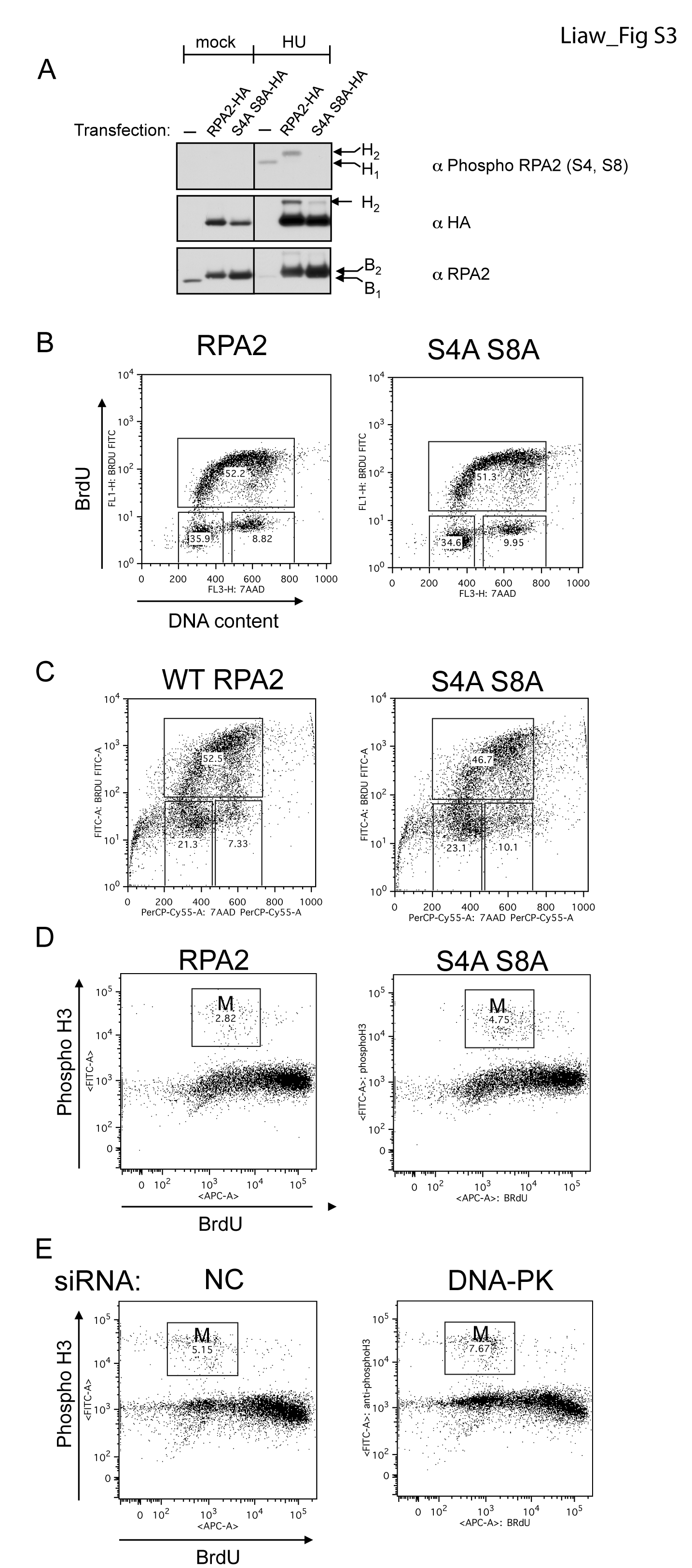

Supplement: Figure S3 — S4A S8A mutations in RPA2 cause higher frequency of mitotic entry. (A) RPA2 S4A, S8A mutant is not hyperphosphorylated in response to DNA damage. Cells were treated with HU in condition described in “C”. B1 and B2 are unmodified endogenous and transfected HA-tagged RPA2, respectively. H1 and H2 are hyperphosphorylated endogenous and transfected HA-tagged RPA2, respectively. (B) Cells expressing either wild type RPA2 or S4A S8A mutant RPA2 (S4A S8A) did not show any distinct differences in cell cycle profiles. (C) Cells expressing S4A S8A mutatant RPA2 entered mitosis more frequently after release from DNA replication stress by HU. Cells were treated with HU for 22 hours and washed to release into media having Nocodazol without HU to inhibit another round of cell cycle. After 11 hours incubation, cells were pulse-labeled with BrdU for one hour before FACS analysis. (D) Mitotic cells positive in both BrdU and Phospho H3 were increased in cells expressing S4A S8A RPA2 mutant. (E) Silencing the expression of DNA-PKcs increased mitotic cells that were positive in both BrdU and Phospho H3. (TIF) [file pone.0021424.s003.tif]

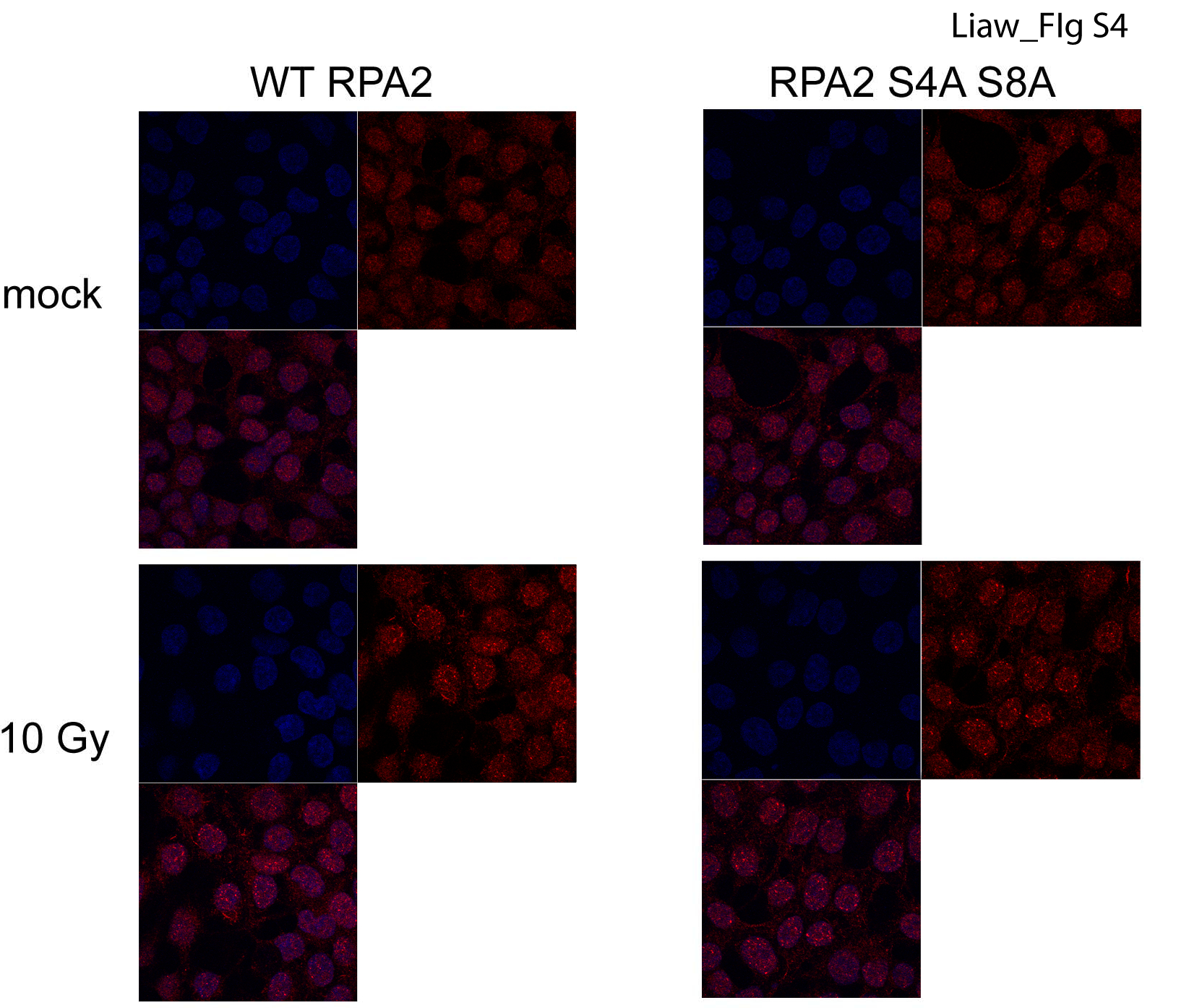

Supplement: Figure S4 — S4A S8A mutations in RPA2 increased the number of RAD51 foci in response to 10 Gy γ-irradiation. (TIF) [file pone.0021424.s004.tif]
